# Case Report: C3 deficiency in two siblings

**DOI:** 10.3389/fped.2024.1424380

**Published:** 2024-07-24

**Authors:** Agustín Bernacchia, Alejandra Ginaca, Sabrina Rotondo, María Pilar Tejada, Daniela Di Giovanni

**Affiliations:** Servicio de Inmunología, Hospital de Niños Ricardo Gutierrez, Buenos Aires, Argentina

**Keywords:** C3 deficiency, recurrent infections, intravenous immunoglobulin, complement system, B-lymphocyte subsets

## Abstract

The complement system, a vital component of innate immunity, consists of various proteins and pathways crucial for the recognition and elimination of pathogens. In addition, it plays a major role in the initiation of adaptive response through the opsonization of antigens, contributing to B-cell activation and memory maintenance. Deficiencies in complement proteins, particularly C3, can lead to severe and recurrent infections as well as immune complex disorders. Here, we present a case report of two siblings with total C3 deficiency resulting from compound heterozygous mutations in C3 (NM_000064.4): c.305dup; [p.Asn103GlnfsTer66] and c.1269 + 5G>T, previously unreported in C3-related diseases. Both, the index case and her sister, presented a history of recurrent infections since early childhood and one of them developed hemolytic uremic syndrome (HUS). Immunological evaluation revealed absent plasma C3 levels, decreased memory B cells, hypogammaglobulinemia, and impaired response to polysaccharide antigens. The siblings showed partial responses to antimicrobial prophylaxis and vaccination, requiring intravenous immunoglobulin replacement therapy, resulting in clinical improvement. Genetic analysis identified additional risk polymorphisms associated with atypical HUS. This case highlights the importance of comprehensive genetic and immunological evaluations in complement deficiencies, along with the potential role of immunoglobulin replacement therapy in managing associated antibody defects.

## 1 Introduction

The complement system, a highly conserved cascade, plays a major role in innate immunity. Comprising over 40 soluble and membrane-bound proteins, this system is activated through three initiation routes: classical, lectin, and alternative pathways. These pathways converge at the cleavage and activation of C3 and subsequently have common steps, highlighting the pivotal role of C3 in the system (1). The activation products of C3 execute multiple effector functions, including opsonization, recruitment, and activation of inflammatory cells that lead to the cytotoxic destruction of microbial pathogens. In addition, they facilitate the effective clearance of pathogens, apoptotic cells, cell debris, and immune complexes (ICs). Through its capacity to modulate B-cell response via co-stimulation, enhance antibody response, and support immunological memory, the complement system bridges the innate and adaptive immunity (2).

C3 deficiency represents a rare autosomal-recessive inherited inborn error of immunity (IEI), characterized by severe and/or recurrent infections and immune complex disorders like rheumatic and renal disease, particularly with childhood onset. Infections typically manifest early in life and are predominantly due to encapsulated bacteria such as *Streptococcus pneumoniae*, *Haemophilus influenzae*, or *Neisseria meningitidis*. Conversely, autoimmune manifestations are less frequent with an older median age of onset (3). Impaired antigen opsonization in C3-deficient individuals is linked to impaired dendritic cell differentiation, memory B-cell responses, and regulatory T-cell development (4). Consequently, patients with C3 deficiency can exhibit a diminished antibody response to polysaccharide antigens and impaired B-cell differentiation; however, extensive immunological investigation has not been addressed in previous reports (5).

Here, we expand the clinical and immunological spectrum of total C3 deficiency by describing the first Argentinian family with a novel genetic variant causing total C3 deficiency.

## 2 Case description

**Figure 1:**
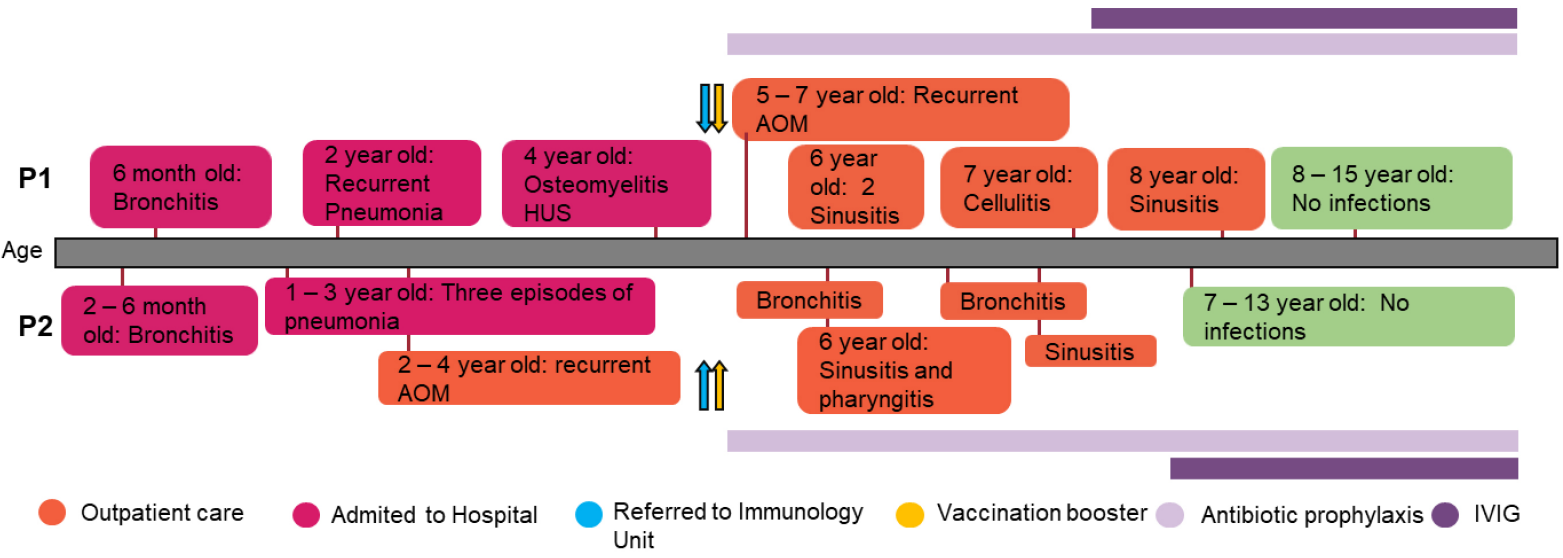
Patients’ clinical records. Graphical display of the clinical course and the treatment administered in the patients.

The index case (P1) is a 15-year-old Argentinian girl, born to healthy, non-consanguineous parents. At the age of 6, she was referred to our immunology unit in Hospital de Niños “Ricardo Gutiérrez” due to a history of persistent and severe infections from early childhood onward. Since she was 4 months old, she has suffered multiple episodes of bronchial obstruction, acute gastroenteritis, sinusitis, and acute otitis media (AOM). At 2 years of age, she was hospitalized due to an episode of pneumonia, requiring intravenous antibiotic treatment. Two years later, she was admitted to the hospital because of an episode of osteomyelitis in the ankle with good response to clindamycin. Three months later she was hospitalized again with bloody diarrhea and acute kidney injury (initial assessment showed: 22% hematocrit; 13.000/mm^3^ platelets; 1.4 mg/dl creatinine). It was assumed to be an episode of hemolytic uremic syndrome (HUS) with requirement of blood transfusion but with no need to start dialysis. Shiga-toxin-producing *Escherichia coli* was not investigated at that moment to determine if it was a typical or atypical HUS (Figure 1).

Initial laboratory tests were unremarkable except for an abnormal serum protein capillary electrophoresis showing an absent beta 2-globulin protein fraction. The immunological work-up for suspected inborn errors of immunity revealed normal lymphocyte subpopulations and Immunoglobulin levels; however, IgG4 levels were undetectable. Antibody response to tetanus toxoid and Rubella vaccine were normal. C3 serum protein levels and classical and alternative complement pathway activity were undetectable on more than one occasion, while C4 levels were near normal (Table 1). Her sister (P2), aged 12 years, also suffered from multiple respiratory tract infections since she was 2 months old, including four hospital admissions due to pneumonia, although no etiological agent was documented. At 5 years of age, immunological work-up revealed hypogammaglobulinemia with normal response to protein, normal lymphocyte subpopulations, absent beta 2-globulin protein fraction in capillary electrophoresis, and absent C3 serum levels as well as undetectable classical and alternative pathway activity. Since the age of eight, she suffered sporadic mild thrombocytopenia (range: 130,000–140,000/mm^3^) without signs of splenomegaly and bleeding (Supplementary Table 1). Furthermore, when she was 11 years old, she developed leukopenia with an absolute neutrophil count of 1.100/mm^3^, which was detected during a routine laboratory evaluation. Cytopenias were assumed to be autoimmune-mediated; however, no autoantibodies against platelets were measured to test this hypothesis. As these were slightly abnormal laboratory findings without accompanying clinical symptoms, no specific treatment has been initiated for these conditions.

**Table 1:** Immunological work-up.

	P1 (6 years old)	P1 (10 years old)	P2 (5 years old)	P2 (8 years old)	II.1	II.2	II.3	I.1
IgG (mg/dl)	1,040 (718–1,608)	1,150^a^ (711–1,743)	578 (682–1,572)	872^a^ (718–1,608)	—	—	—	—
IgA (mg/dl)	196 (78–223)	141 (11–315)	95 (52–150)	79 (78–223)	—	—	—	—
IgM (mg/dl)	243 (83–173)	190 (11–239)	199 (63–177)	232 (83–173)	—	—	—	—
IgE (IU/ml)	615 (<90)	—	112 (<90)	—	—	—	—	—
IgG subclasses (mg/dl)	IgG1 574 (300–840)	—	IgG1 411 (300–840)	—	—	—	—	—
	IgG2 126 (70–255)		IgG2 110 (70–255)					
	IgG3 174 (17–97)		IgG3 107 (17–97)					
	IgG4 ND (2–116)		IgG4 ND (2–116)					
Tetanus IgG (IU/ml)	3.4 (>0.1)	—	1.4 (>0.1)	—	—	—	—	—
Pneumococcal polysaccharide IgG (mg/ml)^b^	<30 (>147)	—	>270 (>147)	—	—	—	—	—
C3 (mg/dl)	<6 (90–150)	<6 (90–150)	<6 (90–150)	<6 (90–150)	47 (90–150)	54 (90–150)	63 (90–150)	72 (90–150)
C4 (mg/dl)	12 (15–35)	11 (15–35)	11 (15–35)	19 (15–35)	27 (15–35)	14 (15–35)	21 (15–35)	23 (15–35)
CH50 (UH50/ml)	<50 (180–280)	<50 (180–280)	<50 (180–280)	—	250 (180–280)	133 (180–280)	204 (180–280)	224 (180–280)
AH50 (minutes)	>60' (<12')	>60 (<12’)	>60' (<12')	—	11' (<12')	12' (<12')	—	—
Lymphocytes (10^3^/mm^3^)	3.9 (1.5–6.5)	3.2 (1.5–6.5)	4.5 (1.5–6.5)	2.7 (1.5–6.5)	—	—	—	—
CD3+ T cells (%)	77 (65–72)	77 (65–72)	72 (67–75)	78 (67–75)	—	—	—	—
CD4+ T cells (%)	34 (33–43)	34 (33–43)	42 (32–48)	42 (32–48)	—	—	—	—
CD8+ T cells (%)	35 (25–32)	38 (25–32)	27 (22–29)	28 (22–29)	—	—	—	—
CD19+ B cells (%)	12 (10–16)	9 (10–16)	17 (11–18)	18 (11–18)	—	—	—	—
CD56+ NK cells (%)	11 (10–19)	12 (10–19)	5 (6–14)	3 (10–19)	—	—	—	—

Immunological laboratory studies in the index patient and her family members. Age-matched reference values are in parentheses. ND, not detectable.

^a^ Under IVIG treatment.

^b^ Pneumococcal vaccine response was measured using the overall response to all 23 serotypes present in the pneumococcal vaccine (PSV23) after 45 days of vaccination, using an enzyme immunoassay kit.

Complement analysis of her parents and other family members revealed some individuals with low serum levels of C3; however, functional assays were normal or slightly diminished (Table 1), and only one family member experienced recurrent infections.

The siblings were suspected to have total C3 deficiency and were booster vaccinated with the conjugated 13-valent pneumococcal vaccine (PCV13) followed by the 23-valent pneumococcal vaccine (PSV23) after eight weeks to prevent severe infections due to *S. pneumoniae*. Forty-five days later, pneumococcal response to polysaccharide antigens was measured. The index case was found to have an impaired antibody response (<30 mg/ml), while her sister had normal levels (>270 mg/ml). In addition, they were booster vaccinated with the quadrivalent meningococcal vaccine (ACYW), and started on antimicrobial prophylaxis with amoxicillin 25 mg/kg once a day. Despite these preventive measures, the patients continued to have recurrent infections, mostly AOM and sinusitis diagnosed by clinical criteria and imaging. These infections were treated orally with amoxicillin–clavulanic acid. However, the failure of antimicrobial prophylaxis to prevent recurrent sinopulmonary infections, as well as the evidence of impaired antibody synthesis or response, led to the decision of initiating intravenous immunoglobulin (IVIG) replacement therapy, leading to a marked decrease in the number of infections.

Subsequent immunological studies revealed normal or elevated plasma levels of alternative complement pathway proteins and regulators such as Factor I, Factor H, properdin, and Factor B. Moreover, the activity of activators of the alternative pathway of complement system such as nephritic factor autoantibody (C3nef), the quantification of C3 activation products (CAP), and soluble membrane attack complex (sC5b9) levels were normal. Taken together, these findings ruled out a possible secondary C3 deficiency due to dysregulation (Table 2). In addition, we detected low levels in C1q; however, subsequent laboratory studies over time revealed fluctuating levels, particularly in association with infectious events, reaching normal values (Supplementary Table 2).

**Table 2:** Complement evaluation.

	P1^a^	P2^a^	Age-matched RV
C1q (mg/L)	61	90	115–240
C2 (mg/L)	26	21	14–29
CFB (mg/L)	461	521	229–394
CFH (mg/L)	650	702	329–557
CFI (mg/L)	20	21	15–34
CAP	Negative	Negative	Negative
C3nef	Negative	Negative	Negative
sC5b9 (ng/ml)	266	328	136–385

Complement system evaluation in the index case and her sister. CAP; complement activation products. RV; reference values.

^a^ The sample was taken during an infectious intercurrence.

**Figure 2:**
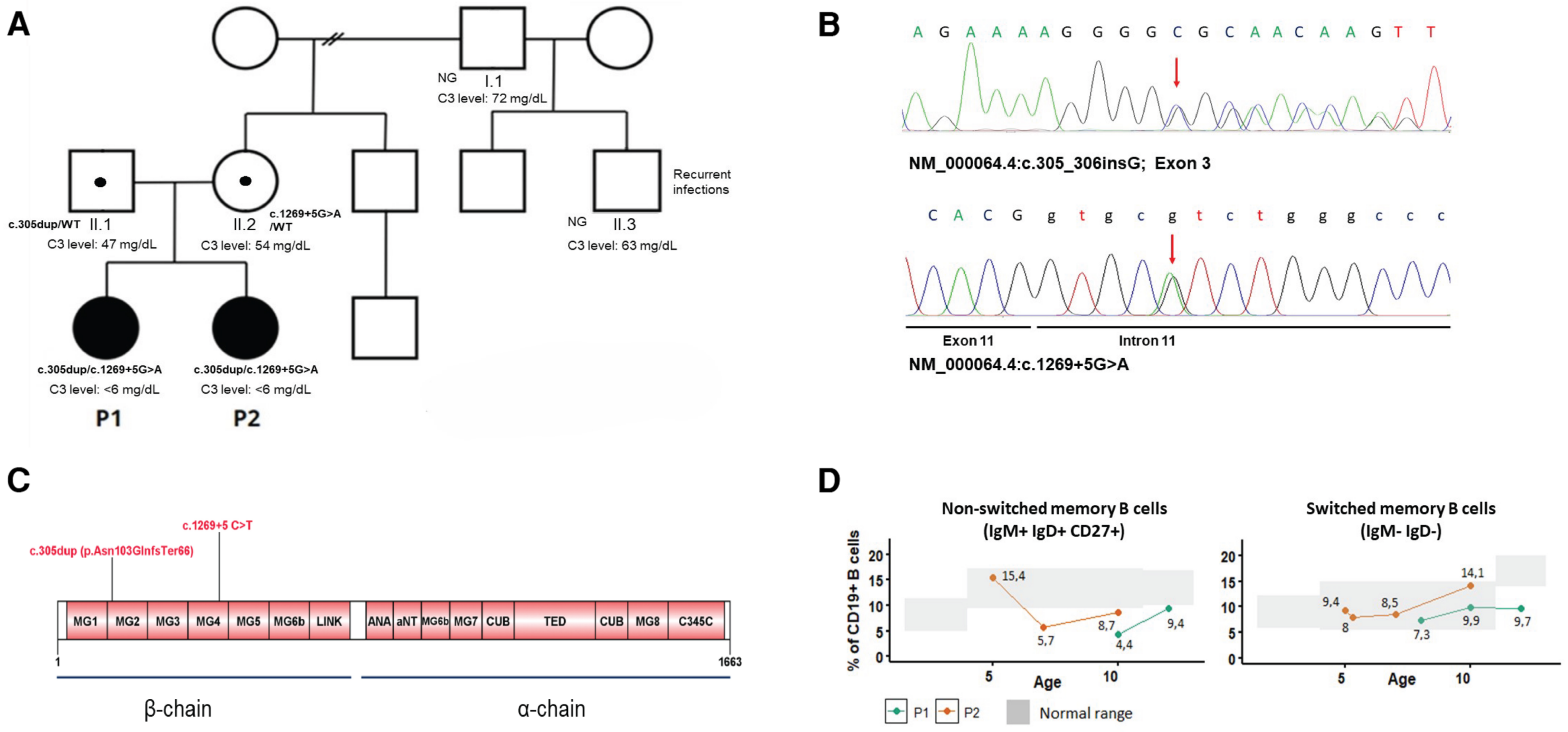
(**A**) Family pedigree and C3 levels of investigated individuals. Filled-in squares/circles represent affected individuals. NG, not genotyped. (**B**) Electropherograms of *C3* mutations in P1. (**C**) Graphical display of C3 protein domains and location of the mutations. (**D**) Level of switched and non-switched memory B cells over time.

Genetic analysis in the index case was performed by next-generation sequencing (NGS) using a gene panel [CFH, MCP (CD46), CFI, CFB, C3, THBD, DGKE, CFHR1, CFHR2, CFHR3, CFHR4, CFHR5, CFP, ADAMTS13] and copy number analysis of *CFH* and *CFHR1-5* by multiplex ligation probe amplification (MLPA). The NGS panel revealed two variants in *C3* (NM_000064.4):c.305dup; [p.Asn103GlnfsTer66] and c.1269+5G>T, not previously reported in C3-related disease. Sanger sequencing of the family demonstrated the compound heterozygous inheritance and the presence of both variants in P2 (Figure 2A). The c.305dup variant is observed at an extremely low frequency in gnomAD v4.0. It occurs in the exon 3 of the protein and is predicted to cause a frameshift leading to a premature stop codon. Therefore, it is predicted to disrupt normal protein functioning either by protein truncation or nonsense-mediated decay. The c.1269+5 G>A variant occurs in the 5′ splice site of intron 11; it is also extremely rare in public databases and is absent from the literature. The computational predictor SpliceAI lookup gives a score of 0.71, which is above the threshold of 0.2, evidence that correlates with an abnormal splicing and impairment of *C3* function. Both variants occur in the β chain of C3 protein and are predicted to undergo nonsense-mediated decay (Figure 2C). The variants were classified as pathogenic and likely pathogenic, respectively, according to the ACMG/AMP guidelines. In addition, other polymorphisms were identified in P1, who was found to be heterozygous for the *CFHR1/CFHR3* gene deletion and for the MCPggaac atypical HUS allele risk, also in the heterozygous state.

To further characterize the immunological phenotype of the patients, we analyzed the presence of autoantibodies and B-cell subpopulations. Antinuclear antibodies were negative in both; however, P1 had positive anti-FH (157 AU, cutoff 100 AU) and anti-C1q antibodies (40 AU, cutoff 20 AU). Flow cytometry analysis of B-cell subpopulations on several occasions showed persistent decreased total memory B cells due to reduced switched (IgD− IgM−) and non-switched memory B cells (CD27+ IgM+ IgD+) in one of the siblings and decreased non-switched memory B cells in her sister (Figure 2D).

Currently, both patients show good evolution and continue with antimicrobial prophylaxis and IVIG treatment, having sporadic bronchial obstruction of out-of-hospital management.

## 3 Discussion

IEI affecting the complement system are considered uncommon, comprising approximately 5% of all IEI (6). Primary total C3 deficiency is an extremely rare autosomal-recessive disease, with fewer than 50 cases reported worldwide since the first C3-deficient patient was described in 1972 (7, 8). Here, we described the clinical and molecular characterization of two siblings with total C3 deficiency, who presented with early-onset severe bacterial infections, and a severe episode of HUS in one of them. Despite antimicrobial prophylaxis, they continue to suffer from recurrent sinopulmonary infections. C3-deficient individuals typically suffer from recurrent pyogenic infections due to encapsulated bacteria such as *S. pneumoniae, H. influenzae, or N. meningitidis* manifesting early in life. IC-related diseases including systemic lupus erythematosus (SLE)-like illness and renal diseases are also observed in this condition.

In peripheral blood, naive and memory B cells can be differentiated by the expression of CD27, IgD, and IgM markers on their surface. Typically, switched memory B cells are IgD− IgM− and are generated in germinal centers by T-B collaboration. Non-switched memory B cells are CD27+ IgM+ IgD+ and their origin is proposed to be germinal center independent, participating in T-independent responses and in the protection against infections by encapsulated bacteria (9, 10). B-cell response is amplified through interaction of opsonizing C3-fragments with complement receptors CD21/CR2. This leads to increased B-cell receptor (BCR) signaling when C3d-opsonized antigens are present on the B-cell surface, facilitating the differentiation from naive to antigen-experienced memory B cells (11).

This pivotal role is supported by the fact that C3-deficient patients have shown altered humoral homeostasis, compromising Immunoglobulins or B cells. Previous reports have described C3-deficient patients with low levels of total IgG or IgG2 and/or IgG4 deficiency (12–16). Moreover, C3 deficiency has also been associated with defective dendritic cell differentiation, altered memory B-cell responses to antigens, and decreased memory B cells (5, 17). Consistent with previous findings, we observed reduced memory B cells, hypogammaglobulinemia, impaired response to polysaccharide antigens, and undetectable levels of IgG4. Collectively, these observations reinforce the role of C3 on B-cell memory generation and potentially explain why these patients continued to have recurrent infections despite antimicrobial prophylaxis. Unfortunately, we were not able to confirm IgG4 deficiency, as we could not measure IgG subclasses on more than one occasion before IVIG therapy administration.

Molecular characterization of C3 deficiency has been described in a few families (3). Here, we expand the genetic spectrum of C3 mutations by reporting two deleterious variants not previously associated with C3-related diseases, resulting in absent plasma C3 levels, which was evidenced also by the absent beta 2-globulin protein fraction, and undetectable classical and alternative complement pathway activity. We also noted decreased levels of C1q and C4, alongside elevated levels of CFB and CFH, probably linked to underlying infectious or inflammatory events. Subsequent measurements ruled out a deficiency in C1q and C4.

Additional polymorphisms were found in heterozygous in P1 that are associated with atypical HUS: the deletion of *CFHR1/CFHR3* genes and the MCPggaac haplotype (18, 19) (Supplementary Table 3). It remains unclear whether these risk polymorphisms may have contributed to the development of renal manifestations in P1 as well as the development of a-FH autoantibodies. Considering that we only analyzed a subset of genes, we cannot rule out the presence of other clinically significant variants in the siblings. The potentially different repertoire of mutations and polymorphisms in complement proteins, the complotype, and their variable penetrance, might explain the difference in clinical observations between the sisters (20). With the increasing availability of next-generation sequencing technologies, investigating the complotype could aid in predicting individualized risk of different clinical manifestations.

As in most autosomal-recessive diseases, heterozygous carriers usually remain asymptomatic and are identified through a history of affected family members. Nevertheless, in some cases, carriers have developed clinical symptoms (21). In this case, further investigation of C3 levels in the family identified a family member with constantly decreased C3 levels similar to those observed in the parents, who are known carriers, and recurrent infections. Although we did not perform confirmatory genetic analysis, this underscores the importance of family testing in complement deficiencies, as the heterozygous state could increase the risk for infections or autoimmunity in certain individuals (22).

Regarding the management of complement deficiencies, clinical control, vaccination with protein-polysaccharides conjugate, and antimicrobial prophylaxis are considered cornerstones in treatment (23); however, in this case, antimicrobial prophylaxis and vaccination proved insufficient to ameliorate the number of infections and, given their antibody defects, IVIG treatment was initiated, resulting in a favorable clinical response.

In summary, to our knowledge, this is the first Argentinian reported case of complete C3 deficiency. We broaden the clinical and genetic spectrum of C3 deficiency by reporting two mutations previously unreported in C3-related diseases. This case highlights the importance of evaluating B-cell subpopulations, IgG subclasses, and IgG responses to polysaccharide antigens in C3-deficient patients to tailor appropriate management strategies. Prompt recognition of complement deficiencies that impair B-cell immunity allows early treatment installation when needed and prevents more severe infections.

## Data Availability

The original contributions presented in the study are included in the article/Supplementary Material, further inquiries can be directed to the corresponding author.
