# Supplementary material for: Case Report: C3 deficiency in two siblings
Source: Front Pediatr. 2024 Jul 24;12:1424380. doi: 10.3389/fped.2024.1424380 (PMC11303290; doi:10.3389/fped.2024.1424380)
Supplement: Supplementary file 1 [file Table1.docx]

**Supplementary table 1: Complete blood count**

| **P2 (Ref. range)** | **sep/2023** | **oct/2022** | **nov/2021** | **nov/2020** | **jun/2020** | **sep/2019** |
| --- | --- | --- | --- | --- | --- | --- |
| **Leukocytes (4.5-13.5)** | 4.2 | 3.2 | 4.6 | 5.4 | 5.5 | 5.9 |
| **Lymphocytes (1.5-6.5)** | 2.3 | 1.7 | 2.6 | 3.0 | 3.4 | 2.7 |
| **Neutrophils (1.5-8.0)** | 1.4 | 1.1 | 1.5 | 1.7 | 1.5 | 2.5 |
| **Platelets (150-450*10^3^)** | 140 | 135 | 150 | 154 | 141 | 141 |
| **Hemoglobin (11.5-15.5)** | 11.9 | 11.9 | 12 | 12.6 | 12.5 | 12.6 |

Complete Blood Count of P2 Showing Mild Intermittent Thrombocytopenia Over Time
