# Supplementary material for: Case Report: C3 deficiency in two siblings
Source: Front Pediatr. 2024 Jul 24;12:1424380. doi: 10.3389/fped.2024.1424380 (PMC11303290; doi:10.3389/fped.2024.1424380)
Supplement: Supplementary file 2 [file Table2.docx]

**Supplementary table 2: Classical pathway protein levels**

| **Date** | **P1** | | | **P2** | | |
| --- | --- | --- | --- | --- | --- | --- |
|  | **C4  (14-29 mg/dL)** | **C1q (115-240 mg/dL)** | **C2 (14-29 mg/dL)** | **C4  (14-29 mg/dL)** | **C1q (115-240 mg/dL)** | **C2 (14-29 mg/dL)** |
| **4/20/2015** | 12 | 61 | 26 | 11 | 90 | 21 |
| **6/25/2015** | 9 | 70 | 21 | 9 | 47 | 17 |
| **2/1/2016** |  |  |  |  |  |  |
| **5/23/2016** | 13 |  |  | 11 |  |  |
| **9/15/2016** | 13 | 118 | 22 | 10 | 155 | 23 |
| **2/2/2017** |  |  |  | 9 | 115 | 17 |
| **6/29/2017** | 13 |  |  |  |  |  |
| **11/16/2017** | 14 |  |  |  |  |  |
| **10/4/2018** |  |  |  | 13 |  |  |
| **1/14/2019** |  |  |  |  |  |  |
| **8/1/2019** |  |  |  | 19 |  |  |
| **9/24/2019** | 11 | 134 | 20 |  |  |  |

Protein Levels of the Classical Pathway in Siblings Over Time
