# Supplementary material for: Case Report: C3 deficiency in two siblings
Source: Front Pediatr. 2024 Jul 24;12:1424380. doi: 10.3389/fped.2024.1424380 (PMC11303290; doi:10.3389/fped.2024.1424380)
Supplement: Supplementary file 3 [file Table3.docx]

**Supplementary Table 3: List of variants detected with the NGS panel in P1.**

| **Chromosome Position** | **Gene** | **RNA Accession** | **CDS** | **Chr** | **Reference Nucleotide** | **Genotype** | **SNP db_xref** | **aa Change** |
| --- | --- | --- | --- | --- | --- | --- | --- | --- |
| 196654324 | CFH | NM_000186.3 | 7 | 1 | A | CC | rs1061147 | p.A307A |
| 196659237 | CFH | NM_000186.3 | 9 | 1 | C | TT | rs1061170 | p.H402Y |
| 196682947 | CFH | NM_000186.3 | 10 | 1 | G | AA | rs2274700 | p.A473A |
| 196686918 | CFH | NM_000186.3 |  | 1 | G | GA | rs6677604 | c.1936+2019G>A |
| 196696933 | CFH | NM_000186.3 |  | 1 | G | AA | rs1410996 | c.2477-543G>A |
| 196712586 | CFH | NM_000186.3 | 20 | 1 | C | CT | rs61822181 | p.T1046T |
| 196713196 | CFH | NM_000186.3 |  | 1 | C | CT | rs147973159 | c.3550+438C>T |
| 196714600 | CFH | NM_000186.3 |  | 1 | C | CA | rs10801560 | c.3551-347C>C |
| 196714670 | CFH | NM_000186.3 |  | 1 | T | TA | rs10801561 | c.3551-277T>T |
| 196757392 | CFHR3 | NM_021023.5 | 4 | 1 | C | TT | rs61735322 | p.S159S |
| 196759960 | CFHR3 | NM_021023.5 |  | 1 | T | CC | rs10922120 | c.883+603T>C |
| 196789161 | CFHR1 | NM_002113.2 |  | 1 | C | GC | rs147050959 | c.172+129C>G |
| 196789200 | CFHR1 | NM_002113.2 |  | 1 | A | AG | rs424772 | c.172+168A>G |
| 196789636 | CFHR1 | NM_002113.2 |  | 1 | T | GG | rs80003882 | c.172+604T>G |
| 196789744 | CFHR1 | NM_002113.2 |  | 1 | A | GG | rs61818876 | c.172+712A>G |
| 196789823 | CFHR1 | NM_002113.2 |  | 1 | G | TT | rs2454299 | c.172+791G>T |
| 196790396 | CFHR1 | NM_002113.2 |  | 1 | T | TC | rs12403900 | c.172+1364T>C |
| 196790415 | CFHR1 | NM_002113.2 |  | 1 | T | TC | rs12403914 | c.172+1383T>C |
| 196790430 | CFHR1 | NM_002113.2 |  | 1 | A | AG | rs12403654 | c.172+1398A>G |
| 196790437 | CFHR1 | NM_002113.2 |  | 1 | T | TA | rs77973645 | c.172+1405T>A |
| 196790448 | CFHR1 | NM_002113.2 |  | 1 | A | AG | rs77473530 | c.172+1416A>G |
| 196790588 | CFHR1 | NM_002113.2 |  | 1 | A | AG |  | c.172+1556A>G |
| 196790599 | CFHR1 | NM_002113.2 |  | 1 | G | GA |  | c.172+1567G>A |
| 196790606 | CFHR1 | NM_002113.2 |  | 1 | T | TC | rs79911321 | c.172+1574T>C |
| 196790616 | CFHR1 | NM_002113.2 |  | 1 | A | AG | rs495141 | c.172+1584A>G |
| 196790629 | CFHR1 | NM_002113.2 |  | 1 | C | CT | rs687948 | c.172+1597C>T |
| 196790691 | CFHR1 | NM_002113.2 |  | 1 | G | GA | rs138388455 | c.172+1659G>A |
| 196790829 | CFHR1 | NM_002113.2 |  | 1 | G | GT | rs1830959 | c.172+1797G>T |
| 196796184 | CFHR1 | NM_002113.2 |  | 1 | G | AA | rs399507 | c.544+49G>A |
| 196796240 | CFHR1 | NM_002113.2 |  | 1 | T | AA | rs438781 | c.544+105T>A |
| 196796361 | CFHR1 | NM_002113.2 |  | 1 | C | TT | rs432234 | c.544+226C>T |
| 196796526 | CFHR1 | NM_002113.2 |  | 1 | A | GG | rs417843 | c.544+391A>G |
| 196796889 | CFHR1 | NM_002113.2 |  | 1 | T | insC | rs67409555 | c.545-312_545-311insC |
| 196797238 | CFHR1 | NM_002113.2 | 4 | 1 | C | TT | rs425757 | p.H157Y |
| 196797244 | CFHR1 | NM_002113.2 | 4 | 1 | C | GG | rs113811987 | p.L159V |
| 196797292 | CFHR1 | NM_002113.2 | 4 | 1 | G | CC | rs388862 | p.E175Q |
| 196797357 | CFHR1 | NM_002113.2 | 4 | 1 | A | GG | rs76835795 | p.T196T |
| 196797845 | CFHR1 | NM_002113.2 |  | 1 | A | TT | rs436234 | c.721+469A>T |
| 196801042 | CFHR1 | NM_002113.2 | 6 | 1 | G | TT | rs4230 | p.R302R |
| 196801078 | CFHR1 | NM_002113.2 | 6 | 1 | A | TT | rs61743621 | p.R314R |
| 196874356 | CFHR4 | NM_001201550.2 | 3 | 1 | G | TT | rs10801578 | p.E125D |
| 196876458 | CFHR4 | NM_001201550.2 | 5 | 1 | A | AG | rs7417769 | p.N210S |
| 196886770 | CFHR4 | NM_001201550.2 |  | 1 | T | CT | rs4915559 | c.1678-570T>C |
| 196913197 | CFHR2 | NM_005666.2 |  | 1 | C | GG | rs9427632 | c.135+129C>G |
| 196913695 | CFHR2 | NM_005666.2 |  | 1 | T | TC | rs138114450 | c.135+627T>C |
| 196914866 | CFHR2 | NM_005666.2 |  | 1 | G | GT | rs1830959 | c.135+1798G>T |
| 196920148 | CFHR2 | NM_005666.2 | 3 | 1 | C | CT | rs4085749 | p.C140C |
| 196920178 | CFHR2 | NM_005666.2 |  | 1 | C | TC | rs3828032 | c.507+20C>T |
| 196920299 | CFHR2 | NM_005666.2 |  | 1 | T | TA | rs3790414 | c.507+141T>A |
| 196922374 | CFHR2 | NM_005666.2 |  | 1 | T | insTTAT | rs66683821 | c.507+2246_507+2247insTTTA |
| 196947030 | CFHR5 | NM_030787.3 |  | 1 | T | TC | rs12755054 | c.186+178T>C |
| 196947139 | CFHR5 | NM_030787.3 |  | 1 | C | CT | rs12731209 | c.186+287C>T |
| 196947314 | CFHR5 | NM_030787.3 |  | 1 | T | CT | rs1170880 | c.186+462T>C |
| 196950350 | CFHR5 | NM_030787.3 |  | 1 | G | GA | rs928440 | c.187-1665G>A |
| 196954225 | CFHR5 | NM_030787.3 |  | 1 | C | CA | rs1750311 | c.558+958C>A |
| 207956559 | CD46 | NM_002389.4 |  | 1 | G | GA | rs1962149 | c.1164-78G>A |
| 207959665 | CD46 | NM_002389.4 |  | 1 | A | AG | rs859705 | c.1302+638A>G |
| 207967719 | CD46 | NM_002389.4 |  | 1 | T | TC | rs7144 | c.*897T>A |
| 207924906 | CD46 | NM_002389.4 |  | 1 | G | GA | rs2796267 | c.-652G>A |
| 207925192 | CD46 | NM_002389.4 |  | 1 | G | GA | rs2796268 | c.-366G>A |
| 110678925 | CFI | NM_000204.3 | 7 | 4 | T | CC | rs11098044 | p.T300A |
| 110685687 | CFI | NM_000204.3 |  | 4 | G | AG | rs79375065 | c.690+6G>A |
| 31914935 | CFB | NM_001710.5 | 3 | 6 | A | AG | rs145955166 | p.R150R |
| 31918464 | CFB | NM_001710.5 | 13 | 6 | A | AG | rs4151659 | p.K565E |
| 136289374 | ADAMTS13 | NM_139025.3 |  | 9 | T | TC | rs2285489 | c.690+6G>G |
| 136290672 | ADAMTS13 | NM_139025.3 | 4 | 9 | G | GA | rs28571612 | p.P118P |
| 136291063 | ADAMTS13 | NM_139025.3 | 5 | 9 | C | CT | rs3118667 | p.A140A |
| 136301982 | ADAMTS13 | NM_139025.3 | 12 | 9 | C | CG | rs2301612 | p.Q448E |
| 136301999 | ADAMTS13 | NM_139025.3 | 12 | 9 | C | CT | rs148365271 | p.T453T |
| 136304497 | ADAMTS13 | NM_139025.3 | 15 | 9 | A | AG | rs3124768 | p.T572T |
| 136308542 | ADAMTS13 | NM_139025.3 | 19 | 9 | C | CT | rs3124767 | p.G760G |
| 136324239 | ADAMTS13 | NM_139025.3 | 29 | 9 | C | CA | rs1055432 | p.T1407T |
| 54921494 | DGKE | NM_003647.2 | 2 | 17 | A | CC | rs3760158 | p.T193T |
| 6677989 | C3 | NM_000064.2 | 41 | 19 | G | AA | rs17030 | p.P1632P |
| 6679511 | C3 | NM_000064.2 |  | 19 | C | TT | rs2277984 | c.4519-4C>T |
| 6690742 | C3 | NM_000064.2 |  | 19 | A | GA | rs11569510 | c.3453-4A>G |
| 6690744 | C3 | NM_000064.2 |  | 19 | A | GA | rs11569509 | c.3453-6A>G |
| 6690746 | C3 | NM_000064.2 |  | 19 | A | GA | rs11569508 | c.3453-8A>G |
| 6696597 | C3 | NM_000064.2 |  | 19 | G | AG | rs2287845 | c.2925+7G>A |
| 6697406 | C3 | NM_000064.2 | 21 | 19 | A | GG | rs423490 | p.A915A |
| 6702157 | C3 | NM_000064.2 | 19 | 19 | C | CG | rs428453 | p.V807V |
| 6702598 | C3 | NM_000064.2 |  | 19 | G | AA | rs406514 | c.2308-8G>A |
| 6709848 | C3 | NM_000064.2 | 14 | 19 | C | CT | rs2230204 | p.V564V |
| 6710782 | C3 | NM_000064.2 | 13 | 19 | G | GT | rs2230203 | p.P518P |
| 6712263 | C3 | NM_000064.2 |  | 19 | C | TC | rs368263824 | c.1269+5G>T |
| 6713262 | C3 | NM_000064.2 | 9 | 19 | G | GA | rs1047286 | p.P314L |
| 6718386 | C3 | NM_000064.2 | 3 | 19 | C | insC | rs773140441 | p.N103QfsTer66 |
| 6718387 | C3 | NM_000064.2 | 3 | 19 | G | GC | rs2230199 | p.R102G |
| 47483800 | CFP | NM_002621.2 | 9 | X | G | AG | rs1048118 | p.N428N |

**(reference genome (GRCh17/hg19)**

In yellow, the MCPggaac haplotype according to (*Esparza-Gordillo J, Goicoechea de Jorge E, Buil A, et al. Predisposition to atypical hemolytic uremic syndrome involves the concurrence of different susceptibility alleles in the regulators of complement activation gene cluster in 1q32 [published correction appears in Hum Mol Genet. 2005 Apr 15;14(8):1107]. Hum Mol Genet. 2005;14(5):703-712. doi:10.1093/hmg/ddi066)* In orange, C3 likely pathogenic and pathogenic variants.
